# Endurance Capacity, Not Body Size, Determines Physical Activity Levels: Role of Skeletal Muscle PEPCK

**DOI:** 10.1371/journal.pone.0005869

**Published:** 2009-06-12

**Authors:** Colleen M. Novak, Carlos Escande, Susan M. Gerber, Eduardo N. Chini, Minzhi Zhang, Steven L. Britton, Lauren G. Koch, James A. Levine

**Affiliations:** 1 Endocrine Research Unit, Mayo Clinic, Rochester, Minnesota, United States of America; 2 Anesthesiology Research, Mayo Clinic, Rochester, Minnesota, United States of America; 3 Department of Physical Medicine and Rehabilitation, University of Michigan, Ann Arbor, Michigan, United States of America; University Paris 7, France

## Abstract

Some people remain lean despite pressure to gain weight. Lean people tend to have high daily activity levels, but the source of this increased activity is unknown. We found that leanness cannot be accounted for by increased weight-corrected food intake in two different types of lean rats. As previously reported in lean people, we found that lean rats had higher daily activity levels; lean rats also expended more energy. These lean rats were developed through artificial selection for high aerobic endurance capacity. To test whether our findings extended to a human population, we measured endurance capacity using a VO_2max_ treadmill test and daily activity in a group of non-exercising individuals. Similar to lean rats selectively bred for endurance capacity, our study revealed that people with higher VO_2max_ also spent more time active throughout the day. Hence, endurance capacity may be the trait that underlies both physical activity levels and leanness. We identified one potential mechanism for the lean, active phenotype in rats, namely high levels of skeletal muscle PEPCK. Therefore, the lean phenotype is characterized by high endurance capacity and high activity and may stem from altered skeletal muscle energetics.

## Introduction

It is thought that the modern increase in obesity is due to the combination of our “thrifty” genes with an obesogenic environment [1], [2]. Why, then, do some people seem to have little trouble staying lean? Unlike obesity, the traits of the minority of individuals who remain lean in the face of environmental pressure to gain weight are often ignored. During selective breeding for diet-induced obesity, for example, lean diet-resistant rats, rather than obesity-prone rats, appear to be unusual compared to the founder population [3]. Likewise, when fed excess calories, some people are less susceptible than others to weight gain [4]. What is different about these rats or people that allows them to resist weight gain? Focusing on the attributes of leanness may lead to fresh insights into the obesity epidemic.

Weight gain can result from increased energy intake and/or decreased energy expenditure. Though overfeeding increases body weight, do lean individuals necessarily have to eat less than obesity-prone people to remain lean? To answer this question, we first investigated voluntary caloric intake in lean and obese rats.

When examining the role of energy expenditure of obesity, the two largest components of total daily energy expenditure receive the most attention: (1) resting or basal metabolic rate, and (2) energy expenditure of activity. Whether or not basal metabolic rate (BMR) is lower in obesity-prone people is a subject of some contention [5]–[10]. Even if BMR is diminished in obesity-prone people, this does not fully account for the positive energy balance in these individuals [6]. We know that daily activity levels are high in lean people compared to obese people [11]. Obese people spend on average over two extra hours sitting compared to lean people [11], [12]. It has been put forward that physical activity and the associated energy expenditure may be key traits that distinguish individuals who are resistant to obesity [4], [13]. Though increasing daily activity is sure to increase energy expenditure, it could be argued that the low activity seen in obesity is secondary to the heightened body mass [14]. To examine this question and probe the source of the heightened activity in lean individuals, we measured daily activity and energy expenditure in a rat model of leanness. We know that rats bred for resistance to obesity on a high-fat diet [15] have high activity levels compared to both diet-induced obese rats and control rats [3], [16]. To determine if high activity is consistently associated with leanness, we measured activity in another group of rats derived through artificial selection. Rats selectively bred for high intrinsic aerobic endurance capacity are lean, whereas their low-endurance counterparts are overweight and prone to metabolic syndrome and cardiovascular disease [17]–[19]. We hypothesized that the lean phenotype is characterized by high endurance and high activity levels. Thus, we focused on these two traits when searching for a biological mechanism underlying the lean phenotype.

It is known that lean people spend more time each day physically active than obese people [11], [12]. The source of this effect is a subject of some debate. It appears that at least some of this difference in daily activity is independent of body mass [11], [12]. We used the information gleaned investigating lean rats to extrapolate potential sources that could underlie the differences in daily activity between lean and obese people. Once we identified this source, namely innate running endurance, an interesting potential mechanism presented itself—one that may underlie the tendency to be active, innate endurance, and leanness.

## Results and Discussion

### Lean rats do not eat less

**Figure 1:**
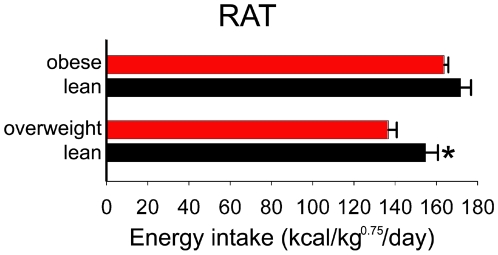
Lean rats do not eat less. Daily caloric intake, corrected for metabolically-active body mass, was not greater in obese compared to lean rats on a high-fat diet (top). Lean, high-endurance rats consumed more calories after mass correction compared to overweight, low-endurance rats (bottom). *p<0.05

To investigate the lean phenotype, we measured food intake in different exemplars of leanness. First, in rats that were selectively bred for resistance to obesity on a high-fat diet [15], the lean rats did not eat fewer calories than the obese rats after correction for body weight (Figure 1). Second, the same was true for another strain of rats that are lean, namely rats selectively bred for high running endurance [18]; the lean rats ate significantly more calories than their overweight counterparts that were selected for low running endurance (Figure 1). Thus, the lean phenotype is not characterized by low caloric intake in rats. This is not to say that weight gain in a given obese individual is not due in part to high food intake, just that the positive energy balance would quite possibly persist even if caloric intake were maintained at the level of a lean individual. This should not be surprising given that obesity has increased even in populations where diet quality has improved and fat intake has decreased over several decades [20], [21].

### Those with high endurance capacity are more active

**Figure 2:**
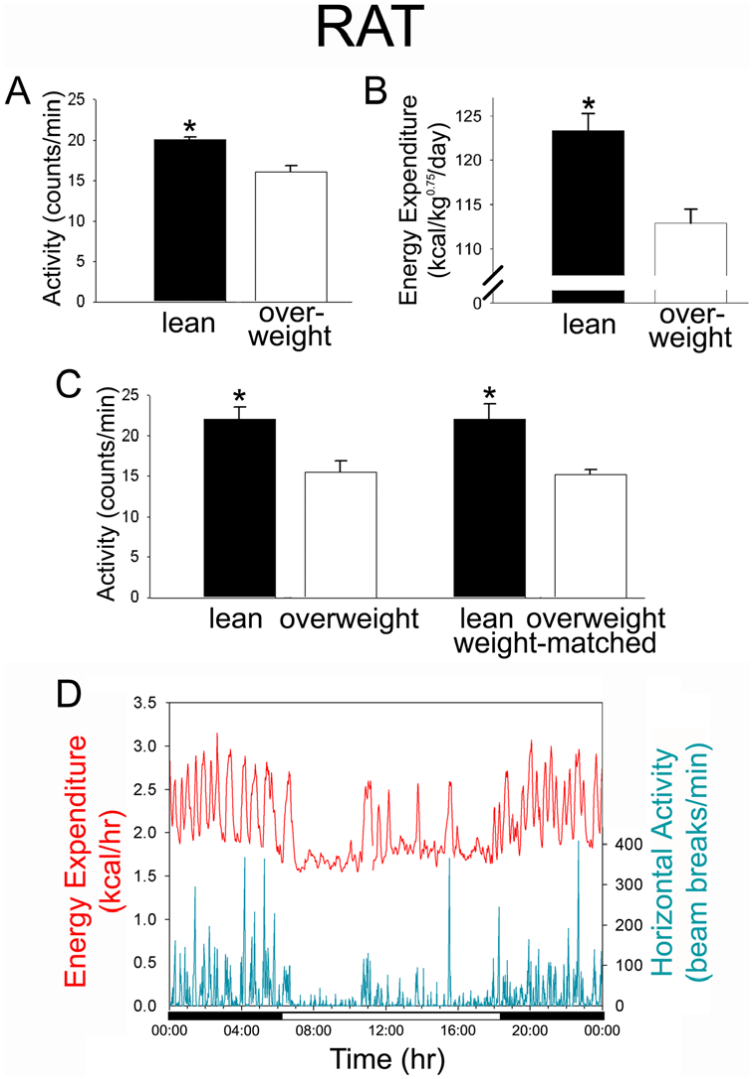
High-endurance rats were more active, regardless of body weight. Physical activity, in beam breaks/min over 24 hrs (mean±SE), was greater in lean, high-endurance rats compared to overweight, low-endurance rats (A), as was body mass-corrected energy expenditure (B). The same effect was seen in female high- and low-endurance rats, even when body weight did not differ between the two groups (C). Elevations in energy expenditure were seen at the same time as peaks in horizontal physical activity (lean rat shown here; D). *p<0.05.

Since leanness cannot be fully explained by differences in food intake, we focused on how calories are burned. It is known that lean animals [3], [16] and lean people [11] consistently have high daily activity levels, but the physiological mechanisms underlying differences daily activity are ill-defined. We measured daily energy expenditure and physical activity in lean, high-endurance rats. The lean rats were 25% more active (Figure 2A) and 25% more ambulatory (Table 1); minute-by-minute, they were active during an additional 64 minutes of the day compared to the overweight rats (Table 1). In humans, this reported effect is even greater, with lean people spending two extra hours standing or walking per day compared to obese people [11], [12]. In the present study we found that this additional activity was reflected in higher weight-corrected energy expenditure in the lean rats (Figure 2B). Lean, high-endurance rats were also more active than overweight rats even when body weights were the same (Figure 2C), demonstrating that high activity in the lean phenotype is not secondary to low body mass. Therefore, in the lean phenotype characterized by high intrinsic running endurance, heightened daily physical activity is not a consequence of a small body mass but rather inherent to the individual's physiology.

**Table 1:** Activity in lean, high-endurance and overweight, low-endurance male rats.

(mean±SE)	Lean	Overweight
Body weight (g)	329±11*	487±15
Ambulation (counts/min)	7.2±0.2*	5.8±0.3
Non-Ambulatory Activity (counts/min)	12.9±0.3*	10.4±0.4
Vertical Activity (counts/min)	0.77±0.01*	0.50±0.06
1-min bins containing activity (% min)	57%±1%*	52%±1%
Energy expenditure of activity (kcal//kg/hr)	0.826±0.050*	0.698±0.032
Resting energy expenditure (kcal/kg/hr)	5.971±0.118*	4.942±0.094
Resting energy expenditure (kcal//g^0.75^/hr)	4.514±0.065*	4.121±0.059

* Significantly greater in lean rats vs. obese rats, *p*<0.05.

How much does the heightened activity seen in the lean rats contribute to their daily energy expenditure, then? As expected, energy expenditure increased with activity throughout the day in rats, as illustrated in Figure 2D. Moreover, when resting energy expenditure (REE) and energy expenditure of activity (EEA) were calculated according to body weight for each rat, EEA (the rat correlate of human NEAT [22]) was significantly higher in the high-endurance rats (Table 1). In other words, the lean rats used more calories to move a given mass than the overweight, low-endurance rats. This does not take into account potential differences in fuel economy of activity that can also affect daily EEA and contribute to total daily energy expenditure. Resting energy expenditure was also higher in lean compared to overweight rats (Table 1). Using our calculation, EEA is roughly 12% of total daily energy expenditure in both groups of rats. In humans, NEAT comprises a much greater proportion of daily energy expenditure—30% or more [23]. Therefore, by logical extension, additional gains in physical activity will result in greater incremental energy expenditure in a human than in a rodent.

We considered the possibility that whether or not the enhanced physical activity and associated energy expenditure accounted for every extra calorie expended in the lean rats may not be the crucial question. It is possible that high physical activity and high aerobic capacity are key, interrelated features of the lean phenotype. This may allow us to more effectively target the fundamental physiological traits and genetic differences underlying leanness. First, however, it was necessary to establish that this effect generalized to human physiology and behavior and was relevant to human health.

**Figure 3:**
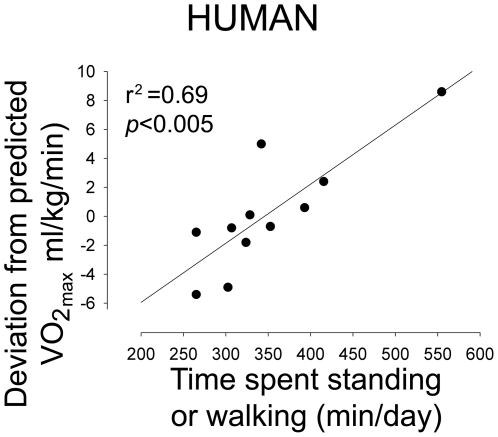
People with higher endurance had higher daily non-exercise activity. VO_2max_ (deviation from predicted VO_2max_ based on age, sex, and body weight) was significantly positively correlated with time spent standing or walking in healthy human volunteers.

If endurance capacity, not body size, is a major factor determining daily activity levels, then the effect we identified in rats should generalize: people with high intrinsic running endurance should also have high daily activity levels. Moreover, if endurance and the tendency to be active are linked at the mechanistic level, then we would expect this association to overshadow the association between daily activity and body weight, as it did in rats (Figure 2). Like spontaneous activity, inborn exercise capacity varies considerably among people, but why some people have higher inborn endurance than others is complex [24]. To test the predictability of our findings from animal studies, we measured VO_2max_ and 10-day baseline physical activity in non-exercising people using a validated Physical Activity Monitoring System [11]. We specifically targeted people who did not engage in regular exercise above the intensity experienced in daily living to rule out the effect of endurance training on VO_2max_. We found that the people who spent more minutes per day standing and walking had higher VO_2max_, normalized to sex, weight, and age (Figure 3). This effect could not be attributed to height, age, sex, weight, adiposity, BMI, or any activity that had the potential to enhance endurance (Table 2). When calculated according to total body mass or fat-free mass, without accounting for sex or the wide range of ages tested, the correlations nearly missed significance (VO_2max_ in ml/kg total body mass/min, *p* = 0.088735; in ml/kg fat-free mass/min *p* = 0.087593).

**Table 2:** VO_2max_ in human participants (Pearson's correlation coefficients).

(mean±SE)	Deviation from predicted VO_2max_	Minutes/day spent Standing or walking
Height (173±3 cm)	0.043	−0.035
Body mass (87±6 kg)	0.033	−0.309
BMI (29±1)	−0.035	−0.374
Fat-free mass (56±3 kg)	0.133	−0.074
Fat mass (31±4 kg)	−0.054	−0.354
Percent body fat (35±3%)	−0.120	−0.357
Age (44±2 years)	0.417	0.073
METS activity (10±3)	0.303	0.229

*r*>│0.467│ for 1-tailed significance of *p*<0.05.

No anthropometric factor accounted for the significant positive correlation between endurance capacity (deviation from predicted VO_2max_) and minutes per day spent standing or walking. The amount of activity over 4 METS per month (1 hr of 5-METS activity = 5) also did not correlate with either VO_2max_ or min/day activity. There were trends toward lower activity levels in those with both higher BMI and higher percent body fat. Note that, in order for any factor to account for the strong association between endurance capacity and activity levels, the factor would have to be positively correlated with both variables or negatively associated with both variables. The correlation between time spent standing and VO_2max_ remained significant when the VO_2max_ deviation was calculated according to fat-free mass instead of total body mass (*r* = 0.56), or if the single individual with unusually high activity levels was removed for the analysis (*r* = 0.64). We also correlated VO_2max_ with activity (min per day spent standing or walking) when VO_2max_ was calculated according to fat-free mass (*r* = 0.440), or total mass (*r* = 0.438), both in ml/kg/min.

Therefore, we conclude that individuals who are more active also have higher endurance capacity. Taken together, these data suggest that high endurance capacity may be a key feature that identifies people who are resistant to obesity and may also hint as to why leanness persists in an obesogenic environment. A relationship between endurance and activity energy expenditure was previously suggested, though usually through the measurement of physical activity energy expenditure using doubly-labeled water and calorimetry (e.g., subtracting resting energy expenditure from total daily energy expenditure)[25]–[27]. Hunter et al. found that women with higher indices of activity also had higher VO_2max_ [27]. Similar results were found in elderly adults: physical activity energy expenditure, which was correlated with physical activity assessed using accelerometers, was positively related to VO_2max_ [25]. In a relatively large study of sedentary older people, VO_2max_ was higher in both men and women who were more active compared to those who were less active [26].

The relationship between VO_2max_ and physical activity previously reported [25]–[27] is not likely to be secondary to training. While training increases VO_2max_ and fitness in younger and older adults [28], [29], it does not increase total daily activity in older people, mostly likely because of compensatory decreases in everyday activity secondary to exercise fatigue [29], [30]. In our study, we took extra precautions to avoid potential training effects by analyzing data from those who did not exercise regularly; we used a relatively stringent standard (even compared to [26]) and demonstrated that participants' more strenuous activity did not confound the results (see Table 2). Similar to the results reported here, both Brochu et al. and Meijer et al. concluded that it was not exercise or high-intensity physical activity, but rather high levels of moderate or regular “spontaneous” activity that related to high aerobic capacity [25], [26]. It is difficult to determine cause and effect in this relationship: Does high endurance allow for high levels of physical activity? Can high levels of spontaneous activity increase endurance? We considered the possibility that both of these factors—aerobic endurance and the tendency to be highly active—may stem from a third cause. In our search for why some people are more active than others, we focused on mechanisms underlying aerobic endurance capacity.

### Lean rats have high levels of skeletal muscle PEPCK

**Figure 4:**
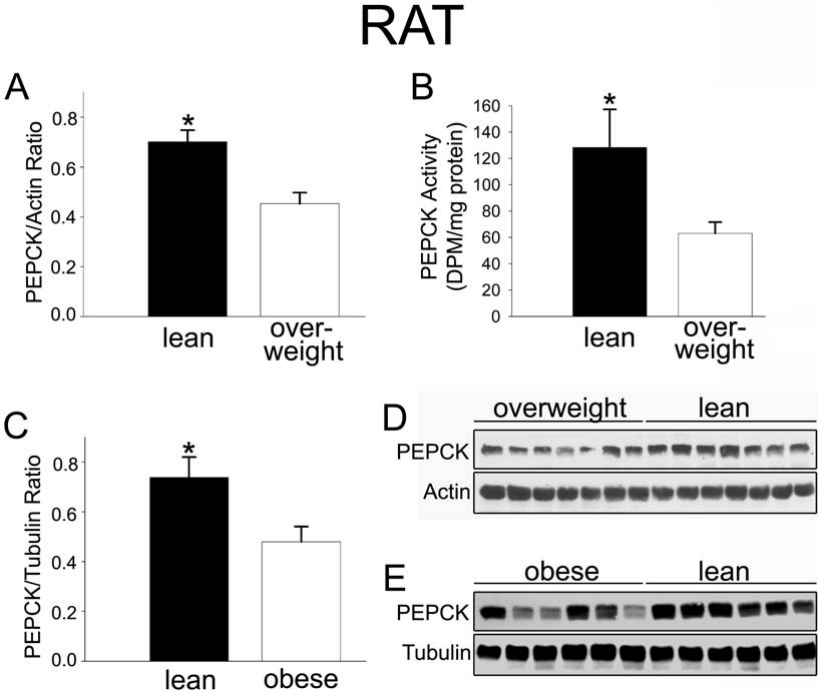
Lean rats have elevated skeletal muscle PEPCK. Lean, high-endurance rats had higher levels of skeletal muscle PEPCK (A,D) and PEPCK enzymatic activity (B) compared to low-endurance, overweight rats. In rats bred for leanness or obesity on a high-fat diet, heightened skeletal muscle PEPCK was also found in lean compared to obese rats (C, E). *p<0.05.

Our data suggest that high aerobic endurance may underlie leanness and high daily activity levels. Recently, these same features were described in mice that express high levels of the enzyme PEPCK-C in skeletal muscle [31]. Hakimi et al. (2008) reported that these mice display several of the traits we see in artificially-selected lean rats: they are lean, long-lived, highly active, behaviorally feisty, have increased caloric intake, and have extremely high running endurance [31], [32]. We therefore measured PEPCK and its enzymatic activity in skeletal muscle. PEPCK levels and enzymatic activity were significantly higher in skeletal muscle from lean, high-endurance rats compared to overweight rats (Figure 4A, B, D). High levels of PEPCK were also found in the muscle of obesity-resistant rats compared to diet-induced obese rats (Figure 4C, E). The presence of elevated skeletal muscle PEPCK in two different sets of lean rats selectively bred for two distinct complex traits—diet resistance and high intrinsic running endurance—implies that high levels of skeletal muscle PEPCK may be a common feature of leanness. Taken together with previous studies [31], [32], our data support the proposition that high muscle PEPCK may be an important element of the lean phenotype.

Because a connection between high activity and high endurance capacity translated to our human participants, this leads us to posit that the mechanisms underlying human leanness should facilitate high stamina, probably by altering skeletal muscle energetics. More specifically, we hypothesize that muscle energy capacity is communicated to the brain to modulate physical activity levels. Moreover, we suspect that this muscle energetic capacity may come at the price of decreased metabolic efficiency [33] and potentially decreased economy of activity as well [34]–[37]. In other words, having high endurance capacity is decidedly un-thrifty. During evolution, food scarcity was only one of several challenges to survival and reproduction. It is entirely conceivable that individuals with high running endurance would have a selective advantage [38](e.g., predator avoidance [39], [40]) and, as our data suggest, these same individuals would have traits favoring resistance to obesity. Our results therefore imply that leanness and high physical activity levels may have resulted as a byproduct during natural selection of high capacity for running endurance. Paradoxically, exploring the mechanisms interconnecting endurance and leanness may be the key to combating obesity.

## Materials and Methods

### 1.1 Human study

#### Ethics Statement

All studies were approved by the Mayo Clinic Institutional Review Board, and all participants gave written informed consent prior to participation in the study.

### 1.1a Daily activity

Eleven non-exercising individuals (6 women and 5 men) completed the 10-day baseline activity measurement using the validated Physical Activity Monitoring System, as previously described [41]. The number of minutes spent sitting, standing, or supine were calculated using inclinometers positioned bilaterally on the torso and legs, and two accelerometers on the back of the hips [41]. Minutes spent standing or walking were averaged over the 10 days for each individual.

### 1.1b VO_2max_

We analyzed activity and VO_2max_ data from individuals who did not engage in regular endurance exercise (less than one hour per week of activity over 4 METS). After a physician screen was completed, 6 women and 5 men were measured for VO_2max_ at the Mayo Clinic Cardiovascular Health Clinic. We used a modified Naughton protocol where the participant walked briskly on the treadmill while speed and incline were altered every two minutes to steadily intensify the effort necessary to continue the test. Oxygen consumption (VO_2_) was measured throughout the study, and relative perceived exertion and blood pressure data were gathered every two minutes. The end of the test was determined by the participant, who was instructed to discontinue walking or running upon reaching exhaustion. The highest single VO_2_ measurement was considered to be the VO_2max_. To ascertain if the VO_2_ obtained was maximal, at least two out of the three following criteria needed to be met: 1. maximum heart rate of ≥165 bpm; 2. exhaustion at the termination of the test (verbal report); and 3. RER≥1. Only one participant was excluded from analysis because criteria were not met; another participant was excluded from the analysis due to recent cessation of lactation. The predicted VO_2max_ was calculated using the following equations: (for males) 60−0.5(age)×body weight (in kg); (for females) 55−0.5(age)×body weight (in kg).

### 1.2 Animal studies

#### Ethics Statement

All animal care was in accordance with institutional guidelines, and all procedures were approved by the Mayo Clinic Animal Care and Use Committee.

Lean, high-endurance capacity (HCR) and overweight, low-endurance capacity (LCR) rats were obtained from Lauren G. Koch and Steven L. Britton [17]–[19] at the University of Michigan. Daily activity and energy expenditure were measured in male (n = 10/group, generation 20) and female (n = 8/group, generation 20) rats; female rats were used in order to minimize the possible confound of body weight and to enable us to examine rats of each phenotype with similar body weights (weight-matched: high-endurance, 256±7 g, range = 240–284 g, n = 5; low-endurance, 265±7 g, respectively; range = 253–175.8 g, n = 3). Food intake and body weight were measured in a second group of lean and overweight male rats (generation 21; high-endurance capacity, n = 8; low-endurance capacity, n = 9). Food intake on a high-fat diet was measured in diet-induced obese (n = 10) and diet-resistant (n = 7) male rats obtained from Charles River [15].

### 1.2a Animal activity, calorimetry, and body composition

Daily activity and energy expenditure were measured using Columbus Instruments small animal indirect calorimeters with Opto-M Varimex Minor activity monitors, which measure horizontal and ambulatory (non-consecutive beam breaks) activity using infrared beams, as previously described [16]. Non-ambulatory activity was calculated as horizontal activity counts minus the ambulatory counts, and we calculated percent time active by determining the number of 1-minute bins during which the rat broke at least a single infrared beam (in any direction or orientation) and divided this number by the total number of minutes measured. We calculated resting energy expenditure (REE) and NEAT, which is the energy expenditure of activity (EEA), for each rat. This was accomplished by identifying which 1-minute bins the rat showed no activity counts. The energy expenditure (in kcal) during minutes when the rat had been inactive for at least 3 min were averaged to obtain REE (in ml/kg^0.75^/hr). The remaining energy expenditure (i.e., total daily energy expenditure–REE) represented EEA. (It should be noted that the thermic effect of food was not measured and not accounted for in these analyses, though this factor is usually very small [23].) The EEA value was then divided by the rat's body weight to yield the energy expended to move one gram of mass (in kcal/kg/hr). After food intake was determined, this group of rats were used to determine body composition using the biochemical method [42].

### 1.2b Skeletal muscle PEPCK

Quadriceps were removed from lean, high-endurance rats and overweight low-capacity rats and snap frozen. Muscle tissue was divided; half of the tissue was used to measure cytosolic phosphoenolpyruvate carboxykinase (PEPCK-C) using Western blot (n = 7/group) and the other half was used to measure PEPCK enzymatic activity [43] (n = 8/group). Liver tissue from starved mice was used as a positive control in the PEPCK activity assay. For diet-induced obese and diet-resistant rats, muscle PEPCK-C levels were determined using Western blot from homogenates of lateral gastrocnemius (n = 7/group).

For the Western analysis, muscle tissue was homogenized in ice-cold RIPA buffer [25 mM Tris-HCl, pH 7.4, 1% Nonidet P-40, 150 mM NaCl, 1% Sodium deoxycholate, 1 mM sodium orthovanadate, 5 mM NaF, and a protease inhibitor cocktail (Roche, Mannheim, Germany)]. Homogenates were incubated at 4°C for 20 minutes under constant agitation. Homogenates were then centrifuged for 10 minutes at 10,000 g at 4°C. Supernatant was collected and its protein concentration determined using the Bradford method (protein assay; Bio-Rad Laboratories, Richmond, CA), with BSA as a standard. Proteins (70 µg/lane) were resolved by SDS-PAGE and transferred by electroblotting onto a PVDF membrane, which was probed with a primary antibody for PEPCK (Cayman Chemical Company, Ann Arbor, MI, USA) and actin (Sigma, St. Louis, MO, USA). Western blots were developed using SuperSignal™ West Pico Chemoluminescent substrate (Pierce, Rockford, IL, USA). Films were scanned and bands quantified by densitometry using Image J (NIH, USA).

### 1.3 Statistical Analyses

Unpaired *t*-tests (1-tailed) were used to compare caloric intake between lean and obese phenotypes, activity and energy expenditure in rats, and PEPCK-C levels and enzymatic activity between groups. When direction of the effect was not predicted, a 2-tailed test was used. A Pearson's correlation coefficient was calculated to determine the relationship between deviation from predicted VO_2max_ and activity levels (min standing or walking per day) in human participants.
